# Role of the Transient Receptor Potential Vanilloid Type 1 (TRPV1) in the Regulation of Nitric Oxide Release in Wistar Rat Aorta

**DOI:** 10.1155/2021/8531975

**Published:** 2021-08-02

**Authors:** Elvira Varela-López, Leonardo del Valle-Mondragón, Vicente Castrejón-Téllez, Israel Pérez-Torres, Araceli Páez Arenas, Felipe Massó Rojas, Verónica Guarner-Lans, Alvaro Vargas-González, Gustavo Pastelín-Hernández, Juan Carlos Torres-Narváez

**Affiliations:** ^1^Laboratorio de Cardiología Translacional, Instituto Nacional de Cardiología “Ignacio Chávez”, Juan Badiano 1, Sección XVI, 14080 Tlalpan, Ciudad de México, Mexico; ^2^Departamento de Farmacología “Dr. Rafael Méndez Martínez”, Instituto Nacional de Cardiología “Ignacio Chávez”, Juan Badiano 1, Sección XVI, 14080 Tlalpan, Ciudad de México, Mexico; ^3^Departamento de Fisiología, Instituto Nacional de Cardiología “Ignacio Chávez”, Juan Badiano 1, Sección XVI, 14080 Tlalpan, Ciudad de México, Mexico; ^4^Departamento of Biomedicina Cardiovascular, Instituto Nacional de Cardiología “Ignacio Chávez”, Juan Badiano 1, Sección XVI, 14080 Tlalpan, Ciudad de México, Mexico

## Abstract

The potential transient vanilloid receptor type 1 (TRPV1) plays important functional roles in the vascular system. In the present study, we explored the role of the TRPV1 in the production of nitric oxide (NO), biopterines (BH4 and BH2), cyclic guanosine monophosphate (cGMP), malondialdehyde (MDA), phosphodiesterase-3 (PDE-3), total antioxidant capacity (TAC), and calcitonin gene-related peptide (CGRP) in the rat aorta. Wistar rats were divided into four groups: (1) control, (2) capsaicin (CS, 20 mg/kg), (3) capsazepine (CZ, 24 mg/kg), and (4) CZ + CS. Treatments were applied daily for 4 days before removing the thoracic aortas for testing of aortic tissue and endothelial cells. TRPV1 activation produced increases in BH4 14%, cGMP 25%, NO 29%, and TAC 59.2% in comparison to the controls. BH2 and MDA increased with CZ. CGRP shows a tendency to decrease with CZ. The analysis by immunocytochemistry confirmed that the TRPV1 is present in aortic endothelial cells. Aortic endothelial cells were obtained from healthy rats and cultured to directly explore the effects of CS and CZ. The activation of the TRPV1 (CS 30 *μ*M) produced increases in BH4 17%, NO 36.6%, TAC 56.3%, and CGRP 65%, when compared to controls. BH2 decreased with CZ + CS. CS effects were diminished by CZ in cells and in the tissue. We conclude that the TRPV1 is a structure present in the membrane of aortic endothelial cells and that it participates in the production of NO. The importance of the TRPV1 should be considered in vascular reactivity studies.

## 1. Introduction

There are numerous membrane structures in the endothelial cells (ECs) that are involved in different biochemical mechanisms, including the synthesis of nitric oxide (NO), and they often participate in the progression of diseases. The synthesis of NO is one of the most studied mechanisms due to its vasodilator action. The pathological role of NO in arterial hypertension, atherosclerosis, aortic aneurysm, and diabetes mellitus, among other pathologies, is due to the chronic or excessive generation of oxidative reactive species (ROS). The disordered production of ROS such as superoxide anion (∙O_2_^−^) and peroxynitrite (ONOO^−^) [1, 2] causes cellular damage by peroxidation of membrane lipids and, as a result, there is an increase in the levels of malondialdehyde (MDA) in tissues [3].

The transient receptor potential vanilloid type 1 (TRPV1) is a membrane structure that was first described in 1997 in nerve endings. It has been mainly studied in pain-related pathologies such as rheumatoid arthritis and migraine [4, 5]. Recently, the TRPV1 has also been described in the ECs of some species [6–8] as a nonselective cation channel which is involved in the permeability of Ca^2+^ [7, 9, 10]. The TRPV1 is activated by thermal stimuli (>42°C), by hemodynamic forces (tangential friction, cellular deformation) [11], by endogenous agonists like anandamide (arachidonylethanolamide), or by exogenous agonists mainly capsaicin (CS) a pungent ingredient of red chilli peppers [4, 5, 10, 12].

Anandamide and CS bind to the TRPV1 through the S512 subunit 3-4 in the cytosol, and the amide group is the receptor-binding region. The action of both molecules is antagonized by capsazepine (CZ) [12].

Once activated, the TRPV1 receptor promotes endothelium-dependent vasodilation through increases in intracellular Ca^2+^ and activation of cofactors such as NADH and tetrahydrobiopterine (BH4) that activate eNOS. On the other hand, CS also activates cannabinoid receptors CB1 and promotes the regulation of Ca^2+^ levels by blocking the L-type Ca^2+^ channels [13–16]. In the presence of *L*-arginine + O_2_, eNOS catalyzes a reaction to produce *L*-citruline and NO. In the smooth muscle, NO activates the soluble guanylyl cyclase which increases cyclic guanosine monophosphate (cGMP) and produces relaxation [14]. Relaxation through the NO pathway is related to the actions of the calcitonin gene-related peptide (CGRP); however, the mechanism is not well known yet. In neurons, the release of CGRP occurs with the activation of TRPV1. This neuropeptide can also be expressed in the endothelium [17–19].

In this work, we evaluated the effects of the activation of TRPV1 in aortic tissue and ECs on parameters involved in the release of NO such as BH4, eNOS, cGMP, phosphodiesterase-3 (PDE-3), CGRP, and total antioxidant capacity (TAC). We also confirmed the presence of the TRPV1 in the ECs of the thoracic aorta of Wistar rats by immunological techniques.

Our hypothesis was that the activation of TRPV1 with CS would promote the modification of the NO levels and of some of the cofactors of its synthetic pathway in the tissue and in the ECs of the aorta.

## 2. Material and Methods

### 2.1. Animals

All procedures in handling animals were approved by the Institutional Ethics Committee according to the National Rules for the care and handling of experimental animals (SAGARPA, NOM-062-ZOO-1999) [20]. Male Wistar rats of 300 to 350 g were kept under standard conditions of temperature and light (12 h light/dark) with a standard diet (Lab diet 5012, PMI Nutrition International, Richmond, IN, USA) and water ad libitum.

### 2.2. Reagents

The pharmacological tools used in this work were of analytical grade (Sigma Chemical Co., St. Louis, MO, USA): capsaicin (8-methyl-N-vanillyl-6-nonenamide), to activate the TRPV1. Capsazepine N-(2-(4-chlorophenyl) ethyl)-1,3,4,5-tetrahydro-7,8-dihydroxy-2H-2-benzazepine-2-arbothioamide] was used as a TRPV1 antagonist.

### 2.3. First Experimental Part

The animals were randomly grouped as follows: (1) control, (2) CS (20 mg/kg), (3) CZ (24 mg/kg), and (4) CZ + CS (first was applied CZ and one hour after was applied CS). All treatments were applied for 4 days before the experiment in daily doses of 5 mg/kg of CS and 6 mg/kg of CZ (subcutaneous). Cs and CZ were diluted in ethanol water.

### 2.4. Determinations in Aortic Tissue

To measure NO, TAC, BH4, BH2, PDE-3, cGMP, CGRP, and MDA levels, the animals were euthanized, and sections of the thoracic aorta were obtained and immediately frozen in liquid nitrogen and stored at -70°C until used. On the day of analysis, tissue samples were homogenized (Polytron PT 1200 E, Kinematica, NY, USA) in cold 100 mM phosphate buffer at pH 7.4, then centrifuged at 16,000 g during 15 min at 10°C (Sorvall SR70, Thermo Scientific Inc., Urbana, IL, USA). Supernatants were filtered through a 0.22 *μ*m nitrocellulose filter (Millipore, Billerica, MA, USA).

The determination of NO and TAC was performed directly on the filtered supernatant, whereas for BH4, BH2, PDE-3, cGMP, and MDA determinations, supernatants were diluted 1 : 10 with 0.1 M NaOH before being analyzed. All samples were kept at -70°C until the beginning of the respective analysis.

### 2.5. Western Blotting: eNOS and TRPV1

Protein extraction from aortic tissue was carried out. Samples of protein (50 *μ*g) were separated by 10% SDS-PAGE for eNOS and TRPV1 and transferred onto polyvinylidene difluoride (PVDF) membranes. After blocking, the membrane was incubated with specific primary monoclonal rabbit anti-TRPV1 (Sigma-Aldrich, V2764) and monoclonal mouse anti-NOS3 (Santa Cruz Biotechnology, sc-376751) antibodies [21]. All blots were incubated as a control with monoclonal mouse anti-*α*-actin (Santa Cruz Biotechnology, sc-3225) antibody overnight at 4°C and then incubated with horseradish peroxidase-conjugated secondary antibodies. Proteins were visualized using the ECL detection system (Bio-Rad) and quantified by densitometry (GS-800 densitometer, Bio-Rad).

### 2.6. Second Experimental Part

Once these results in aortic tissue were obtained, the investigation was directed to study the role of TRPV1 in the regulation of some parameters of the NO synthesis pathway directly in the ECs.

### 2.7. Endothelial Cell Culture

The ECs were isolated from the aorta by digestion with collagenase [19, 22]. The aorta was perfused with a solution of HEPES (1 M HEPES, 2.2 g/L glucose), and ECs were obtained by the action of type II collagenase 0.2% (Gibco Invitrogen Co. Grand Island, NY, USA). Cells were cultured in endothelial growth medium M199 (Gibco Invitrogen Co. Grand Island, NY, USA) with heat inactivated 20% fetal calf serum (Hyclone Thermo, Logan, Utah, USA), 2 mmol/L *L*-glutamine (Sigma Chemical Co., St. Louis, MO, USA), 5 U/mL Heparin (Pisa, Edo. De México, México), 37.5 *μ*g/mL supplement endothelial cell growth, and mixture of antibiotics (penicillin 10,000 units, streptomycin 10 mg, amphotericin B 25 *μ*g/mL) from Sigma-Aldrich (St. Louis, MO, USA). Cells were grown to confluence in a humid environment at 37°C with 7% CO_2_.

Subcultures of ECs were detached from culture flasks using a solution of 0.5 g trypsin in EDTA 0.2 g (Sigma-Aldrich, St. Louis, MO, USA). The cells were previously washed with HEPES solution.

The cells were identified as endothelial at the fourth pass by their characteristic spindle-shape morphology [19], by the positive immunohistochemistry for eNOS [23], and by testing positive to the following markers: CD54 (ICAM), CD106 (VCAM).

### 2.8. Activation of the TRPV1 Directly on Endothelial Cell

ECs were seeded in 6-well culture (Corning Inc., NY, USA), as follows; (1) control and incubated with (2) CS 30 *μ*M, (3) CZ 10 *μ*M, and (4) CZ + CS. Stimulation was performed for 30 minutes at 37°C, with rocking motion on a shaker tray. The supernatant was recovered and stored and frozen at -70°C until the respective measurements were performed. The following parameters were measured: BH4, NO, MDA, TAC, and CGRP. The supernatants were diluted 1 : 10 with cold 50 mM phosphate buffer at pH 7.4 and filtered through a 0.22 *μ*m nitrocellulose filter (Millipore, Billerica, MA, USA). The determination of NO and TAC was performed directly on the filtered supernatant, whereas for BH4 and MDA determinations, supernatants were diluted 1 : 10 with 0.1 M NaOH before being analyzed. All samples were kept at -70°C until the start of the respective analyses. For extraction of the CGRP released and/or soluble CGRP, samples were placed in 10 volumes of 2 M acetic acid and transferred to a boiling water bath for 5 min. Subsequently, the samples were homogenized for 5 min using an Polytron homogenizer (PT 1200 E, Kinematica, NY, USA), and the homogenates were centrifuged at 16,000 g (Sorvall SR70, Thermo Scientific Inc., Urbana, IL, USA) for 60 min at 4°C. Clear supernatants were filtered with Sep-Pak C18 classic cartridges (Millipore, Billerica, MA, USA) and eluted with 60% (*v*/*v*) acetonitrile + 0.1% trifluoroacetic acid as described in Kurts et al. [21] 20% acetonitrile was then added and reserved for later HPLC analysis.

### 2.9. Measurement of Biopterins

The levels of BH4 and BH2 were determined simultaneously by the zone capillary electrophoresis method, with UV-Vis detection by diode array, according to the methodology of Han et al. [24]. The concentration of the biopterins (BH4 or BH2) was determined by a standard curve of 0-100 pmol/mL.

### 2.10. Measurement of Nitric Oxide

NO was quantified in samples according to the Tenorio method [25], under UV-Vis spectrophotometry (Evolution 220, Thermo Scientific, Urbana, Illinois, USA), by wavelength difference (572-587 nm) at room temperature. For this, 100 *μ*L of a solution of vanadium chloride (III) at 0.8% (*w*/*v*) in 1 M phosphoric acid was added to 20 *μ*L of the sample. The reading was performed on a spectrophotometer at a wavelength of 572 and 587 nm, considering for calculations, the difference in absorption of 572 nm-587 nm. The results were expressed in pmol/mL, and the concentration of NO was calculated by using a standard curve of nitrites, in a range of 0-200 pmol/mL.

### 2.11. Measurement of Phosphodiesterase-3

Ion-exchange HPLC analysis of the PDE-3 was carried out as described by Geoffroy et al. [26]. The samples were deproteinized with cold methanol and then with cold 10% trichloroacetic acid, both in a 10 : 1 ratio.

### 2.12. Measurement of CGRP

The CGRP analysis was performed according to Seon et al.'s method [27]. The processed samples were purified by preparative reverse-phase HPLC (ACQUITY UPLC System, Waters Corporation, Barcelona, España) on a Waters RCM compact preparative cartridge Delta-Pak C 18 (300 Å; 25 3 100 mm/Waters, Barcelona, España) eluted at a flow rate of 8 mL/min by a linear gradient of acetonitrile in 0.1% trifluoroacetic acid in water (5 min; wash at 5% acetonitrile followed by a 5–60% linear gradient of acetonitrile at 0.5%/min). The purified sample was then subjected to an HPLC analysis (ACQUITY UPLC System, Waters Corporation, Barcelona, España), using Lichrosorb C18 column (5 mm, 4.6x250 mm/Phenomenex, CA, USA), as well a linear acetonitrile gradient (20-58%) at a flow rate of 0.75 mL/min, and a detection wavelength of 220 nm at 10°C. The concentration of the CGRP was determined by a standard curve of 0-100 fmol/mL.

### 2.13. Immunocytochemistry

The ECs were grown on coverslips in Petri dishes with M199 medium (Sigma-Aldrich St Louis MO) supplemented with 10% FBS (HyClone Thermo-fisher Waltham Mass USA) and an antibiotic-antimycotic mixture (Sigma-Aldrich St Louis MO) for 48 hrs. at 37°C in an atmosphere with 5% CO2. Subsequently, cells were stimulated with CS for 30 minutes. Then, the cells were fixed with a 4% paraformaldehyde solution for 10 minutes at 4°C. The cells were permeabilized with Triton X-100 0.1% (Sigma-Aldrich St Louis MO) at room temperature for 10 minutes, followed by incubation with a rabbit anti-TRPV1 antibody 1 : 50 (Alomone Labs Jerusalem ISR), in a humidified chamber overnight at 4°C. An unlabeled anti-TRPV1 antibody was detected by incubation with an anti-rabbit anti-IGg antibody conjugated with fluorescein isothiocyanate (FITC) (Thermo Fisher Waltham Mass USA). The cells were examined by confocal fluorescence microscopy with an inverted LSM 700 Axio Observer confocal microscope (Carl Zeiss Jena GmbH) at a 40X magnification. We include a 2.5 X digital zoom image in order to appreciate more cell details. DAPI was used to contra-stain the nucleus. The intensity of fluorescence was determined by the zen blue program (Carl Zeiss Jena GmbH).

### 2.14. Statistical Analysis

The statistical analysis used in all cases consisted of a one-way analysis of variance, followed by a Students *t*-test for paired data with *n* = 10 per group for tissue homogenates and *n* = 6 per group for ECs in culture, *p* < 0.05.

## 3. Results

### 3.1. Nitric Oxide and Systemic Oxidative Stress

**Figure 1:**
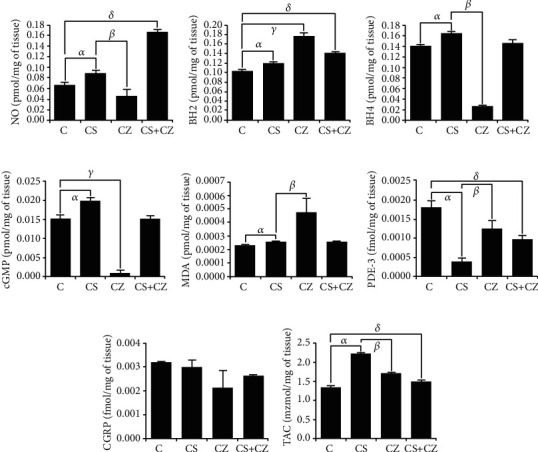
Biochemical determinations of NO (a), BH2 (b), BH4 (c), cGMP (d), MDA (e), PDE-3 (f), CGRP (g), and TAC (h) levels in aortic tissue of control rats (C), with CS, CZ, and with CZ + CS treatments. *n* = 10, *p* < 0.05, *α*: CS vs. C; *β*: CS vs. CZ; *δ*: C vs. CZ + CS.

To evaluate whether NO levels were modified by activation of TRPV1 in the aortic tissue, we used the aortas of rats treated with CS.  Figure 1(a) shows that NO was significantly increased when compared to the control group 22% (0.07 ± 0.004 to 0.09 ± 0.0042 pmol/mg of tissue). The effect of CS was reduced by 44% with the treatment with CZ (0.09 ± 0.004 to 0.05 ± 0.012 pmol/mg of tissue). Surprisingly, the treatment of CZ + CS doubled the NO production when compared to the control (0.07 ± 0.004 to 0.17 ± 0.005 pmol/mg of tissue).

BH4 is a very important cofactor in the synthesis of NO; therefore, it must be in molecular equilibrium with eNOS and ROS to avoid the oxidation of BH4 to BH2.  Figure 1(b) shows the imbalance that occurs in BH2 due to CS. There was an increase of 20% (0.10 ± 0.002 to 0.12 ± 0.002 pmol/mg of tissue) with respect to the control, but it did not significantly affect the production of NO. A significant increase of 80% in the BH2 levels was observed with the CZ treatment (0.10 ± 0.002 to 0.18 ± 0.005 pmol/mg of tissue), and a 40% elevation was found with the treatment with CZ + CS (0.10 ± 0.002 to 0.14 ± 0.0001 pmol/mg of tissue).

Figure 1(c) shows an increase of BH4 of 13% (0.14 ± 0.0017 to 0.16 ± 0.0011 pmol/mg of tissue) with respect to control. The CZ treatment inhibited the effect of CS by lowering BH4 levels in 81% (0.16 ± 0.002 to 0.03 ± 0.001 pmol/mg of tissue) in comparison to the CS group. We did not observe changes in the BH4 levels with CZ + CS.

Similar effects were observed in the cGMP levels (Figure 1(d)). CS produced an increase of 25% (0.015 ± 0.0007 to 0.020 ± 0.0009 pmol/mg of tissue). This effect was inhibited by CZ by 95% (0.020 ± 0.0009 to 0.001 ± 0.0007 pmol/mg of tissue). We did not observe changes with the CZ + CS treatment when compared to the control.

On the other hand, in this study, we evaluated the levels of MDA and PDE-3 as biomarkers of cell damage. CZ increased the levels of MDA (0.0002 ± 6.1*E* − 7 to 0.0005 ± 1.0*E* − 04 pmol/mg of tissue). There was no effect of the treatments with CS and CZ + CS. The levels of PDE-3 were decreased with the three treatments, (0.0004 ± 0.0001) with CS, (0.0012 ± 0.0002) with CZ, and (0.0010 ± 0.0001) with CZ + CS, respectively (in pmol/mg of tissue regard to control 0.0018 ± 0.0002). The CGRP levels were only affected by the treatment with CZ (0.00.2 ± 2.8 *E* − 05 to 0.0021 ± 6.8 *E* − 04 fmol/mg of tissue) when compared to the control (Figure 1(g)). However, the levels did not reach a level of a statistically significant difference. Panel (h) shows the antioxidant increase generated by CS with respect to the control group (1.3 ± 0.041 to 2.2 ± 0.010 mmol/mg of tissue). The effect was inhibited by treatments with CZ to 1.7 ± 0.002 and with CZ + CS to 1.5 ± 0.015 mmol/mg of tissue, respectively.

### 3.2. Enzyme Expression

**Figure 2:**
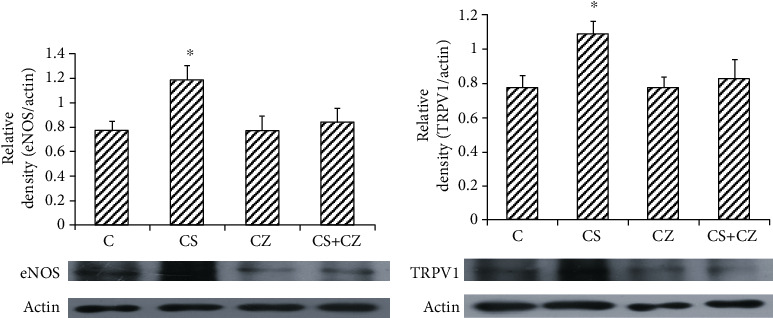
(a) eNOS and (b) TRPV1 expression in aortic tissue. Control (C) and CS, CZ, and CZ + CS treatments. The bars represent mean ± SEM of 4 animals per group. ^#^*p* < 0.05; ^∗^*p* < 0.05 vs. control group. Representative western blot analysis is shown.

There was an increased expression of eNOS (33%) and TRPV1 (30%) in the aortic tissue with the treatment with CS when compared to their expressions in the control group (Figures 2(a) and 2(b)). CZ and the combination of agents had no effect on eNOS and TRPV1 expression.

### 3.3. Identification of the Vanilloid Receptor

**Figure 3:**
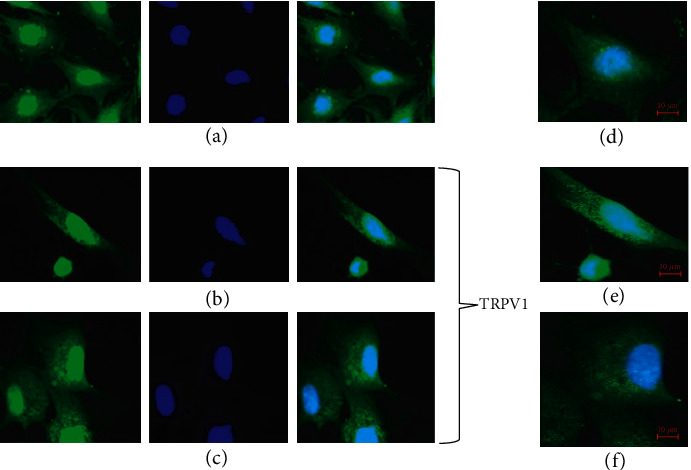
Immunoflourescence of TRPV1 from aortic ECs using a fluorescein-coupled secondary antibody. The cells were treated with buffer (control) (a) or with CS (30 *μ*M, 30 min) (b and c). The image is a confocal section that shows the arrangement of the channel in the cell (green dots) and the nucleus (blue). Image was taken on 20X objective. (c–f) represent an increase or zoom of the figures for a better observation.

The immunofluorescence test showed that CS produced an increase in the specific label that indicates an increase in the expression of TRPV1 on the surface of ECs (Figures 3(b) and 3(c)) with respect to cells without CS (Figure 3(a)). Figures 3(d)–3(f) represent amplifications of the picture. The media of the intensity units of fluorescence was measured with the zen program and corresponded to 4134.24 F.U. for the control without CS (3D) vs. 9606.52 F.U. in 3E and 9843.33 F.U. in 3F with CS.

### 3.4. Endothelial Cells, Nitric Oxide, and Oxidative Stress

The ECs obtained from the aorta were cultured to carry out pharmacological tests directly on the cell membranes and measure the cellular responses in the incubation liquid.

**Figure 4:**
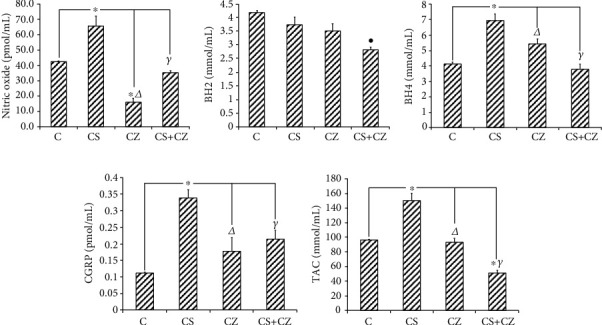
Changes on levels of NO (a), BH2 (b), BH4 (c), CGRP (d), and TAC (e) determined in the proliferation medium of ECs incubated with CS, CZ, and CZ + CS in comparison with ECs incubated in proliferation medium without reactant action and in comparison with CS group. *n* = 6 per treatment. ^∗^C vs. CS; *Δ*CS vs. CZ; ***γ***CS vs. CZ + CS; ●C vs. CZ + CS.

NO (Figure 4(a)) was significantly increased by 36% with respect to the control group (42.4 ± 6.7 to 65.4 ± 6.3 pmol/mL). CZ decreases NO release by 80% in comparison to the CS group (65.4 ± 6.3 to 14.07 ± 1.84 pmol/mL). CZ inhibited the effect of CS in the group where CZ + CS was applied (34.9 ± 1.5 pmol/mL).

The BH2 (Figure 1(b)) only had a significant decrease of 20% with respect to the control when CZ + CS was applied (4.2 ± 0.045 to 2.95 ± 0.12 pmol/mL).

In panel (c) of Figure 4, there is a significant increase in BH4 with CS 41% (4.3 ± 0.2 to 7.3 ± 0.3 pmol/mL), which correlates with the increase in NO with CS in panel (a). The CZ decreased the levels of BH4 in 28% (7.3 ± 0.3 to 5.26 ± 0.24 pmol/mL) when compared to the CS group. A similar effect occurred with the decrease in NO with CZ which can be observed in panel (a). CZ and CS neutralized their effects and BH4 remained at a baseline level. Therefore, the difference to highlight is a 46% decrease with the CS group (7.3 ± 0.3 to 3.9 ± 0.13 pmol/mL).

CGRP is a neuropeptide whose mechanisms of action to produce vasodilation and other cardiovascular effects continue to be studied by different research groups. In Figure 4(d), it is observed that the activation of TRPV1 significantly stimulated the release of CGRP (0.12 ± 0.011 to 0.34 ± 0.02 fmol/mL) in the ECs of the aorta. The CGRP increase by CS was inhibited with the application of CZ (0.34 ± 0.02 to 0.17 ± 0.04 fmol/mL) and with CZ + CS (0.34 ± 0.02 to 0.2 ± 0.03 fmol/mL).

The activation of TRPV1 in the ECs correlates with the increase in NO, BH4, and CGRP and with an improvement in the total antioxidant capacity (Figure 4(d)) of 56% (96 ± 6 to 150 ± 10 mmol/mL). These effects were inhibited by CZ and CZ + CS to 92.9 ± 5 and 51 ± 3.2 mmol/mL, respectively.

## 4. Discussion

The aorta is the largest artery in the vascular system participating in the distribution of oxygenated blood to all the other organs and tissues. It also has other important functions that depend on its elasticity, such as the control of systemic vascular resistance, heart rate, and blood pressure [28–30]. NO is a metabolite that is involved in the regulation of these functions [30]. Our objective in this study was to analyze the participation of TRPV1 in the regulation of NO and the involvement of some cofactors needed for its synthesis in the aorta. We extracted the thoracic aorta to study the vessel's tissue and the endothelial cells. We sought to determine if the synthesis route of NO was modified by the treatment or incubation with CS. We also tried to confirm the presence of TRPV1 in the membrane of the aortic endothelial cell in the Wistar rat.

The activation of TRPV1 in the aortic cells stimulated the production pathway of NO by increasing the expression of eNOS and by elevating the levels of BH4 and cGMP.

With the treatment of animals with CS, the levels of MDA in the aorta were maintained in a similar level as in the control group. Since this signal of damage was not altered, the activation of TRPV1 seems to participate in the control and maintenance of cellular function. On the other hand, the activation of the receptor decreased the levels of PDE-3, preventing this enzyme from catalyzing the hydrolysis of cGMP to inactivate its function.

In addition, CS generated an increase in the TAC, but unexpectedly, the TAC improved in the aortas of rats treated with CZ. This result may be due to the antioxidant characteristics of both molecules. However, further research is needed on this topic.

Derived from these observations and the increase in the expression of eNOS and TRPV1 generated by CS, we decided to undertake a second experimental stage. We explored the participation of TRPV1 in the regulation of the levels of NO, BH2, BH4, CGRP, and TAC in isolated ECs. We also verified if the TRPV1 forms an integral part of the endothelial cell membrane of the Wistar rat aorta and whether it is activated by CS. This is a relevant fact for the experimental research is aimed at understanding endothelial and vascular function, since many studies on vascular diseases in which NO is involved are carried out in this rat strain.

Incubation with CS had a positive effect on the production of BH4, NO, and CGRP in ECs, and it also improved TAC. These results reinforce the importance of TRPV1 in this form of signaling.

The biochemical tests showed changes in the levels of the biomarkers studied and suggest that TRPV1 has regulatory actions on the NO pathway and in the control of the antioxidant enzymatic system. The inhibition of the enzyme that hydrolyzes cGMP was also observed.

Even if the pharmacological tests were carried out in the tissues and cells of healthy animals, alteration in the homeostasis of the aorta or in the endothelial cell was clearly induced. Moreover, our results show that the activation of TRPV1 by CS maintains or increases the levels of NO, BH4, and cGMP in tissue and in endothelial cells and that these effects were diminished by the action of CZ.

## 5. Conclusion

Based on the results obtained with pharmacological and immunocytochemical studies, we conclude that the TRPV1 receptor is an integral protein of the aortic endothelial cell membrane in the rat and that this receptor is involved in the regulation of the NO pathway.

## Data Availability

The data used to support the findings of this study are included within the article.
